# An Entity Relation Extraction Method for Few-Shot Learning on the Food Health and Safety Domain

**DOI:** 10.1155/2022/1879483

**Published:** 2022-02-21

**Authors:** Min Zuo, Baoyu Zhang, Qingchuan Zhang, Wenjing Yan, Dongmei Ai

**Affiliations:** ^1^Beijing Technology and Business University, National Engineering Laboratory for Agri-product Quality Traceability, Beijing 100048, China; ^2^Basic Experimental of Natural Science, University of Science and Technology Beijing, Beijing 100048, China; ^3^School of Mathematics and Physics, University of Science and Technology Beijing, Beijing 100048, China

## Abstract

In recent years, entity relation extraction has been a critical technique to help people analyze complex structured text data. However, there is no advanced research in food health and safety to help people analyze the complex concepts between food and human health and their relationships. This paper proposes an entity relation extraction method FHER for the few-shot learning in the food health and safety domain. For few-shot learning in the food health and safety domain, we propose three methods that effectively improve the performance of entity relationship extraction. The three methods are applied to the self-built data sets FH and MHD. The experimental results show that the method can effectively extract domain-related entities and their relations in a small sample size environment.

## 1. Introduction

Food is inextricably linked to human health, particularly the nutritional components that can significantly improve health. For instance, increased spermidine intake protects against cancer, metabolic disease, heart disease, and neurodegeneration [1]. A higher intake of whole grains and dietary fiber is associated with a decreased risk of death from liver cancer and disease [2]. In addition, contaminants in food or an excessive amount of artificial additives have a detrimental effect on human health and even cause various diseases. For instance, interference with the intestinal microbiota's metabolites caused by food contaminants (polycyclic aromatic hydrocarbons, polychlorobiphenyls, brominated flame retardants, dioxins, pesticides, and heterocyclic amines) may promote the establishment of an inflammatory state in the intestine [3]. Food emulsifiers and thickeners influence the intestinal microbiota, mucosal barriers, and inflammatory pathways, and there are numerous possible pathogenic mechanisms [4].

With the proliferation of available human health data, humans use predictive or classification models to extract useful information, which helps people enjoy better health protection. Typical of this available human health data is the electronic health record (HER), the largest source of medical text data. One of the critical points in analyzing these unstructured textual data is to extract vital medical concepts, so many named entity recognition methods and entity relation extraction methods have emerged. Wan et al. proposed an ElMo-ET-CRF model approach for extracting medical named entities from Chinese electronic medical records (CEMR) using dynamic context-dependent ElMo character embedding to merge more lexical, syntactic, and semantic information that alleviate the long context-dependency problem [5]. Luo et al. proposed a novel tagging scheme considering overlapping relations to solve the overlapping problem in biomedical texts and then built the Att-BiLSTM-CRF model to extract the entities and their relations according to the rules [6]. Fei et al. proposed a new cross-graph neural model for joint extraction of overlapping entity relations in biomedical texts. They treat the entity relation extraction task as a relational triad prediction and construct entity graphs by enumerating possible candidate entity spans [7].

It is possible to help explicitly store the relationships between complex medical concepts for use in subsequent tasks by extracting them. Similarly, for the food health and safety domain, food regulators sample food products on the market and examine the ingredients and form structured data. However, in addition to structured data, more unstructured textual data goes beyond the act of food sampling and inspection to include announcements of food safety events, news reports, and knowledge about food and human health. Cenikj et al. proposed a method to detect the relationship between food and disease entities from text. They explored the feasibility of migration learning using a pretrained model based on BERT and achieved good results with few-shot learning [8]. Popovski et al. proposed a rule engine for extracting food concepts called FoodIE, a rule-based named entity recognition method with rule content describing food entities' computational linguistic and semantic information [9].

However, the food health and safety field does not have many available resources like the biomedical field, so few-shot learning becomes an effective method to improve information extraction. Qu et al. proposed a Bayesian meta-learning method for relation extraction in few-shot learning environment, applying graphical neural networks to global relation graphs to improve accuracy [10]. Sainz et al. reformulated relation extraction as an entailment task with good results for zero- or small-sample relation extraction tasks [11].

The essential technique for extracting valid information in the food health and safety domain is entity relation extraction. This task requires the model to identify entities and relations in a sentence correctly and combine them correctly. The complexity of the task and the small sample size together contribute to the difficulty of the entity relation extraction task with few-shot learning. For supervised deep learning models, small sample size can lead to an underfitting of the model. In other words, there is not enough information to train a valid model. In addition, the model tends to converge at a local optimum, leading to poorer practical results of the model.

To solve the underfitting problem, we propose two methods to enrich the semantic features of the input text. The first is to disassemble Chinese characters into radicals (https://en.wikipedia.org/wiki/Radical_(Chinese_characters)) and construct semantic input at the radical level. As pictographs, radicals are part of the structure of Chinese characters. The radicals have specific meanings; in other words, they represent part of the semantic information of the constituent characters. Therefore, constructing semantic input at the level of radicals can enrich the semantic information of the input text very well [12, 13]. The second approach to enrich semantic information is to fuse input text characters and word vectors. As units with independent meanings, characters are often given new meanings after forming words, and word information can significantly complement the interpretation of words within words [14]. For instance, the Chinese character “瘦 (lean),” “肉 (meat),” and “精 (essence)” form the word “瘦肉精,” which stands for a drug that is banned from being added to animal feed. In their study, Chen and Hu also verified that Chinese words contain rich semantic information [15]. Tran et al. combined the advantages of character- and word-level translation and proposed a new method for translating Chinese into Vietnamese [16].

**Figure 1:**
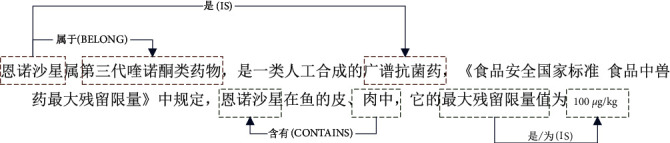
Examples of sentences in the field of food health and safety and the entity relations that exist within it.

To solve the problem of the model falling into local optimality, we propose a text noise removal model to help the model converge quickly to the global optimum. As shown in Figure 1, for a given input text: “Enrofloxacin belongs to the third generation of quinolones, is a class of synthetic broad-spectrum antibacterial drugs, according to the “National Food Safety Standards Maximum Residue Limits for Veterinary Drugs in Food,” the maximum residue limit of enrofloxacin in the skin and meat of fish is 100 *μ*g/kg.” There are four groups of entity relations combinations in the sentence.

We need to extract the two entity relation groups below the sentence in Figure 1 (index values 3 and 4 in Table 1). The two entity relation groups above the sentence (index values 1 and 2 in Table 1) are weakly related to food health and safety. In this paper, we believe that we need to accurately propose the entity relation groups that are food-health-related. The entity relation groups that are weakly related to food health and safety will become noise to affect the extraction effect.

To effectively extract entities and relations for texts in the food health and safety domain in a small-sample learning environment, this paper proposes a method for entity relation extraction in the food health and safety domain, FHER (food health entity relation extraction).

Our contribution can be summarized as follows:

1. To reduce the influence of noise in the text, we propose a text denoising method for domain entity relation extraction to convert the text denoising task into a sequence prediction task, which effectively reduces the correct range of entity relation extraction.
2. To address the lack of semantic information due to the small sample size under the few-shot learning, we propose constructing the input at the part-head level to enrich the semantic information.
3. We propose the fused character and word method to improve the ambiguity problem caused by errors in Chinese word separation boundaries.

## 2. Related Works

There are two approaches in current research [17]. Traditionally, a pipeline approach is used to extract entity mentions using a named entity recognizer and then predict the relationship between each pair of extracted entity mentions [18, 19]. This approach inevitably brings the problem of error propagation. To alleviate the error propagation problem, Yu and Lam proposed a joint extraction approach that performs two subtasks simultaneously [20]. The joint extraction approach is gradually becoming mainstream in entity relation extraction tasks. Geng et al. proposed an end-to-end joint entity and relation extraction method based on a combined attention mechanism of convolutional and recurrent neural networks [21]. Wan et al. proposed a region-based hypergraph network (RHGN) for joint entity and relation extraction, introducing the concept of regional hyper nodes to enhance contextual connections [22]. Wei et al. proposed a cascading pointer labelling approach, which solves the problem of overlapping entity relations [23]. Qiao et al. proposed a joint entity relation extraction model BERT-BiLSTM-LSTM for agriculture and verified that the BERT model has better migration in agriculture [24].

Due to the scarcity of textual data in the food health and safety domain, few-shot learning can effectively improve the effectiveness of entity relation extraction. Most of the current research on small-sample learning has focused on relationship extraction tasks. For relation extraction in a few-shot learning environment, Qu et al. propose a Bayesian meta-learning method that applies graphical neural networks to global relation graphs to improve accuracy [10]. Sainz et al. reformulate relation extraction as an entailment task with good results for zero- or small-sample relation extraction tasks [11].

For the entity relation extraction task in food health and safety, we propose a joint entity relation extraction method FHER with noise removal and feature enhancement, which mainly addresses how to perform effective entity relation extraction in a few-shot learning environment with high noise and low data volume. Our model adopts a cascading pointer annotation approach [23] to mitigate the entity overlap problem in the entity relation extraction task.

## 3. Methodology

### 3.1. Overview

The FHER method divides the whole entity relation extraction task into five parts: the first is a text noise removal model, the second is the construction of a radical level input, the third is the fusion of character and word features, the fourth is the prediction of possible subjects in a sentence, and the last is the prediction of relations and corresponding objects.

**Figure 2:**
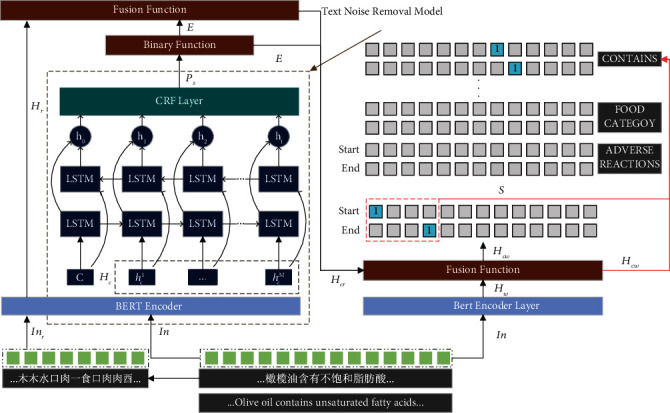
FHER model structure diagram.

Figure 2 shows the overall structure of the model. The input sentence In passes through the BERT Encoder layer to obtain vector *H*_*c*_. The prediction sequence *P*_*s*_ is obtained after passing through the text noise removal model. The position vector *E* can be obtained through the binary function. Then, the input In_*r*_ of sentence In at the radical level is constructed, and the vector *H*_*r*_ is obtained after passing through the BERT encoder layer. The vectors *H*_*c*_ and *H*_*r*_ and the position vector *E* are fed into the fusion function to obtain the vector *H*_*cw*_ after fusing the characters and words. Using the position vector *S* and the fusion vector *H*_*cw*_, we can obtain the possible object positions corresponding to each relation and thus the combination of entity relations present in the sentence.

We define the entity relation extraction task in the food health and safety domain: unstructured input text, output extracted human health-related concepts and their relationships, and further form food- and health-related knowledge triad. Formally, for a given sentence *x*, all possible triples *T*{*s*, *r*, *o*} are extracted, with *s* representing the subject in sentence *x*, *o* representing the object in sentence *x*, and *r* representing the relationship between the subject and the object. For sentence *x*, the probability of all possible triples *T* that it contains is as follows:

(1) $$\begin{array}{c} \prod_{\left(s,r,o\right)\in T}p\left(\left(s,r,o\right)|x\right)=\prod_{s\in T}p\left(s|x\right)\prod_{\left(r,o\right)\in T|s}p\left(\left(r,o\right)|s,x\right), \end{array}$$

where, according to the chain rule, *s* ∈ *T* denotes the subject appearing in the triplet *T*, *T|s* denotes the triplet *T* containing the subject, (*r*, *o*) ∈ *T|s* denotes the combination of objects and relations appearing in the triplet containing the subject, and *R* is the set of relations. For a given training set *D*, the probability that all sentences may contain the triplet *T* is as follows:

(2) $$\begin{array}{c} T=\prod_{i=1}^{D}\left[\prod_{\left(s,r,o\right)\in T_{i}}p\left(\left(s,r,o\right)|x_{i}\right)\right]. \end{array}$$

The goal of the entity relation extraction task in the food health and safety domain is to find all possible triples (*s*, *r*, *o*) in the data set *D*.

### 3.2. BERT Encoder

The implementation of the language model basis of the model is the encoder layer (BERT encoder). The BERT model consists of a multilayer bidirectional transformer consisting of an encoder that learns the valid information in the context very well [25]. We use a pretrained BERT model using a Chinese corpus from the food domain to encode the context of the input sentence In. Formally, for a sentence of length *M* can be represented as In=[*w*_1_, *w*_2_, *w*_3_,…,  *w*_*M*_]. We input these tokens into the same BERT encoder layer as the trained one to obtain a vector representation *H*_*c*_ of the input sentences as follows:

(3) $$\begin{array}{c} H_{c}=\left[h_{c}^{1},h_{c}^{2},h_{c}^{3},\ldots ,h_{c}^{M}\right]=\text{BERTEncoder}\left(\text{In}\right). \end{array}$$

### 3.3. Text Noise Removal Model

**Figure 3:**
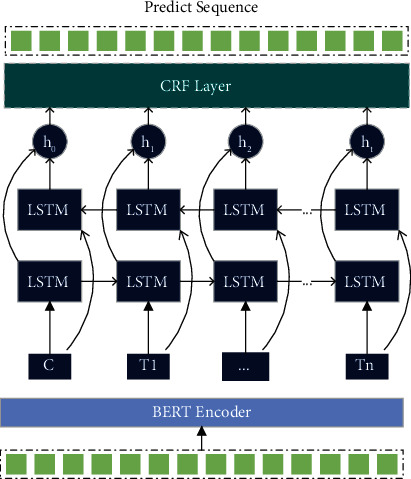
Text noise removal model structure.

We summarized the possible relationships between concepts related to food health and safety into 12 kinds by reading many food health and safety information.  Table 2 lists the details of the relations since the text contains combinations of entity relations with low relevance to the domain; these irrelevant combinations of entity relations act as noise. It will interfere with identifying entity relation combinations in the food health and safety domain. To reduce the influence of noise in the text, we propose a text denoising method for domain entity relation extraction to convert the text denoising task into a sequence prediction task, which effectively reduces the correct range of entity relation extraction.  Figure 3 shows the structure of the text noise removal model structure.

**Figure 4:**
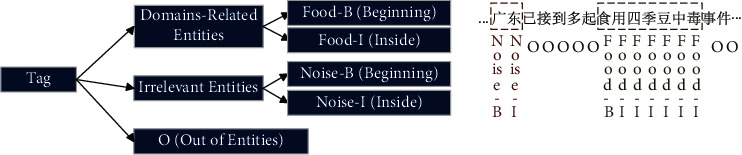
Modified BIO tagging method. The input text characters can be tagged as the beginning and middle positions of the entity and outside the entity. The “Food” prefix indicates that the entity is related to food health and safety, and the “Noise” prefix indicates that the entity is a noise that is not related to food health and safety.

We transformed the text noise removal task into a sequence prediction model. To capture the sentence context information, we used the BiLSTM layer for further feature extraction. Then, considering the dependencies between the labels, we use the undirected probabilistic statistical graphical model CRF layer to handle the exact sequence labelling problem. Each character is assigned a BIO tag (B represents the beginning of an entity, I represents In an entity, and O represents Out of an entity). At the same time, we modified this tagging method to predict the possible types of entities (see Figure 4).

With the BERT encoder, we obtain the vector representation *H*_*c*_ of the sentence, which is then fed to the BiLSTM layer to obtain the intermediate vector representation *H*_*Bi*_ as follows:

(4) $$\begin{array}{c} H_{Bi}=\left[h_{Bi}^{1},h_{Bi}^{2},h_{Bi}^{3},\ldots ,h_{Bi}^{M}\right]. \end{array}$$

For the input sequence In and the corresponding predicted label sequence *Y*, the model is trained to obtain the prediction sequence result *P*_s_ as follows:

(5) $$\begin{array}{c} P_{s}=\left[{\hat{y}}_{1},{\hat{y}}_{2},{\hat{y}}_{3}\ldots ,{\hat{y}}_{M}\right]=\text{CRF}\left(H_{Bi}\right). \end{array}$$

### 3.4. Construction of the Radical Feature

**Figure 5:**
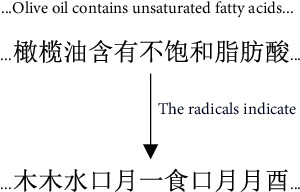
Representing sentences with radicals.

As the smallest semantic unit, radicals themselves have particular semantic meanings. Chinese characters are usually composed of smaller primary radicals, which are the most basic units that constitute the meaning of Chinese characters. In essence, this radical semantic information helps make characters with similar radical sequences (writing order) close to each other in the vector space, so it can be used to enrich the semantic information of the word vector and enhance the model effect. As shown in Figure 5, the sentence “Olive oil contains unsaturated fatty acids” can be represented at the radical level as “木木水口月一食口月月酉.” The radical for the word fat is “月.” When “月” is used as a radical, it can be interpreted as relating to the moon or meat. The radical of the character “酸” is “酉,” and its meaning is related to alcohol and fermentation. The meaning of the decomposed radicals can enhance the semantic information of the word itself.

Formally, for the input sentence In, the corresponding radical input In_*r*_ is generated based on the radical decomposition, and for each character *w*_*ri*_ in In, the radical is added to In_*r*_ if there is a radical, or directly to In_*r*_ if it is a unique word. Finally, we get the input In_*r*_ at the radical level as follows:

(6) $$\begin{array}{c} {\text{In}}_{r}=\left[w_{r1},w_{r2},w_{r3},\ldots ,w_{rM}\right]. \end{array}$$

Similarly, the vector representation *H*_*r*_ corresponding to the input In_*r*_ can be obtained through the BERT encoder layer as follows:

(7) $$\begin{array}{c} H_{r}=\left[h_{r}^{1},h_{r}^{2},h_{r}^{3},\ldots ,h_{r}^{M}\right]=\text{BERTEncoder}\left(In_{r}\right). \end{array}$$

The sequence prediction result *P*_*s*_ obtained from the text noise removal model is converted into a position vector *E* by a binary function *I*(·). *I*(·) will mark the food health-related tag positions as 1 and the irrelevant tag positions as 0.

(8) $$\begin{array}{c} E=\left[e_{1},e_{2},\ldots ,e_{M}\right]=I\left(P_{s}\right). \end{array}$$

The input vectors *H*_*c*_ and *H*_*r*_ and the position vector *E* are input to the fusion function *G*(·). The function *G*(·) multiplies the position vectors *E* with the input vectors and concatenates them. At last, the vector *H*_*cr*_ is calculated as follows:

(9) $$\begin{array}{c} H_{er}=\text{concatenates}\left(G\left(H_{c},H_{r},E\right)\right). \end{array}$$

### 3.5. Character and Word Fusion

As in Section 3.1, we use the BERT encoder layer to get the vector representation of the input sentence *H*_*w*_. The difference is that the BERT pretraining model used in the previous section is to segment the text into Chinese characters before training, while the BERT pretraining model used here is first to segment the text into Chinese words before training. The length of the input sentence after Chinese word separation is *N*.

(10) $$\begin{array}{c} H_{w}=\left[h_{w}^{1},h_{w}^{2},h_{w}^{3},\ldots ,h_{w}^{N}\right]=\text{BERTEncoder}\left(\text{In}\right). \end{array}$$

Because of the difference between Chinese and English word construction methods, different characters will be combined into words in Chinese word construction. Then the generated words will generate new meanings. So, in the food health and safety domain, especially in few-shot learning, we need to use as much prior knowledge as possible to help us improve the recognition, so we fuse the character-based vector representation *H*_*c*_ and the word-based vector representation *H*_*w*_.

We multiply the position vector *E* obtained from the text noise removal model with *H*_*w*_ to obtain the vector *H*_we_. Then we concatenate *H*_*cr*_ to *H*_*w*_ according to the corresponding position to obtain the fused vector *H*_*cw*_ (see equation (11)), where the *K*(·) function expands the vector *H*_*cr*_ to the same length as *H*_*w*_. For example, the vector for the word “食物” is A, and the vectors for the characters “食” and “物” are B and C, respectively. Then, concatenate vectors A and B, and concatenate vectors A and C.

(11) $$\begin{array}{c} H_{cw}=\text{concatenate}\left(G\left(H_{cr}\right)EH_{w}\right). \end{array}$$

After combining characters and words, we can predict the location in the sentence where the subject may be. Because the entity relation extraction task itself suffers from the problem of overlapping entity relations, according to the view proposed by Wei et al. [23]: for a given subject *s*, any relation related to *s* (relations in *T*) corresponds to the corresponding object *o* in the sentence, while all other relations necessarily have no corresponding object in the sentence, that is, the set of corresponding objects is the empty set. So we first predict the possible positions of the subject in the sentence.

For a given input, we predict the location of potential subjects. Two binary classifiers form a subject marker, which marks potential subjects' starting and end positions. The subject marker is expressed as follows:

(12) $$\begin{array}{c} P_{i}^{{\text{sub}}_{\text{head}}}=\sigma \left(K_{\text{head}}H_{cw}^{i}+b_{\text{head}}\right), \end{array}$$

(13) $$\begin{array}{c} P_{i}^{{\text{sub}}_{\text{tail}}}=\sigma \left(K_{\text{tail}}H_{cw}^{i}+b_{\text{tail}}\right), \end{array}$$

where *P*_*i*_^sub_head_^ denotes the probability that each position in the input vector may be the start position of the subject and *P*_*i*_^sub_tail_^ in denotes the probability that each position in the input vector may be the end position of the subject. If this probability is greater than the threshold *τ* set in advance, it is for the position is a candidate for the start or end of the subject, marked as 1, and the rest is marked as 0. *K*_head_ and *K*_tail_ are learnable parameters, and *b*_head_ and *b*_tail_ are deviation parameters.

### 3.6. Relation Extraction

We obtain all possible sets of entity relations in the input sentence by object identification of each subject to all relations. After the subject tagger, we obtain the candidate subject position information, incorporated into the fusion vector as follows:

(14) $$\begin{array}{c} \hat{H}=H_{cw}^{i}+\text{sub.} \end{array}$$

Similarly, the object tagger predicts the potential object location, consisting of two binary classifiers. The object tagger is expressed as follows:

(15) $$\begin{array}{c} P_{i}^{{\text{obj}}_{\text{head}}}=\sigma \left(K_{\text{head}}^{r}\hat{H}+b_{\text{head}}^{r}\right), \end{array}$$

(16) $$\begin{array}{c} P_{i}^{{\text{obj}}_{\text{tail}}}=\sigma \left(K_{\text{tail}}^{r}\hat{H}+b_{\text{tail}}^{r}\right). \end{array}$$

The process of predicting the location of the object is similar to that of predicting the location of the subject. *P*_*i*_^obj_head_^ in equation (15) denotes the probability that each position in the input vector may be the start position of the object, and *P*_*i*_^obj_tail_^ in equation (16) denotes the probability that each position in the input vector may be the end position of the object. *K*_head_^*r*^ and *K*_tail_^*r*^ are learnable parameters, and *b*_head_^*r*^ and *b*_tail_^*r*^ are deviation parameters. All relations in the set *R* have an object tagger to mark the start and end positions of candidate objects.

## 4. Result Analysis and Discussion

### 4.1. Data Set and Evaluation Metrics

This paper uses an independently constructed food health-related data set FH (Food Health Dataset). Twelve types of food-health-related relationships are defined, and then “OTHER” relationship types are defined to represent less relevant relationships to food health in the sample data.  Table 2 lists the 12 relationship types, the Chinese and English names of the included relationships, and their abbreviations. In addition, the paper constructs a small sample data set MHD (Medical Health Dataset) of medical-health-related data to measure the method's generalization performance. The data set MHD consists of 5 relationship types with 200 sentences. The training set contains 180 sentences, and the test set contains 20 sentences.

**Figure 6:**
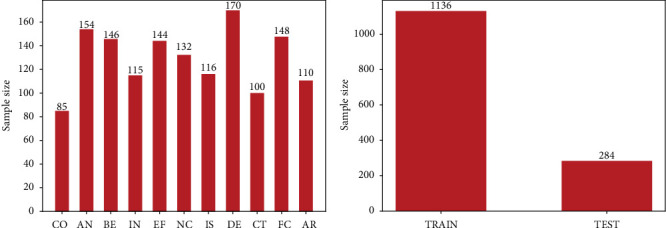
Details of the FH data set show: (a) the number of 11 food health and safety-related relationships and (b) the amount of data in the training and test sets.

The total number of valid annotated sentences in the FH data set is 1,420. This paper divides the FH data set into a training set and a test set.  Figure 6 shows the details of the data set.

To improve the performance of the model, we introduced more data samples from the public domain into the data set FH to form the incremental data set FH++, which is expanded from 1,420 sentences to 11,327 sentences to get better results in the text noise removal model. For the entity relation extraction task, we remove the “Other” relationships from the FH data set to form the FFH (filtered food health data) data set (http://39.96.33.199:9009/FFH_dataset.zip) used for entity relation extraction experiments.

For the evaluation metrics used in the experiments, we used three commonly used evaluation metrics, precision (*P*), recall (*R*), and *F*1 value, to measure the experimental results from different aspects. Their calculation criteria are as follows: in the formula, *TP*_*i*_ indicates that the model correctly predicts the number of relationships present in given sentence In_*i*_, *FP*_*i*_ indicates that the model incorrectly predicts the number of relations that do not exist in given sentence In_*i*_, and *FN*_*i*_ denotes the number of relations that the model failed to predict for given sentence In_*i*_ contained in the sentence.

(17) $$\begin{array}{c} P=\frac{TP_{i}}{TP_{i}+FP_{i}}\\ R=\frac{TP_{i}}{TP_{i}+FN_{i}}\\ F1=\frac{2*P*R}{P+R}. \end{array}$$

For the text-to-noise model, we use the metric *P* to measure whether the model is predicting the location sequence correctly, the metric *R* to measure the model's ability to predict the location of entities in a complete way, and the metric *F*1 to measure model performance in aggregate. We focus more on metric *P* than on metrics *R* and *F*1, as it is more important to find the noise accurately.

For the entity relation extraction task, we use indicator *P* to measure how well the model extracts the correct combination of entity relationships in a sentence, indicator *R* to measure the model's ability to extract coverage in the face of sentences containing multiple relationships, and indicator *F*1 to measure the model's performance in aggregate.

### 4.2. Implementation Details

The pretrained BERT model we implemented in the deep learning framework PyTorch uses the Chinese pretrained BERT model (BERT-wmm) published by Cui et al. [26] with its default hyperparameter settings. The models in this research are implemented on a PC with the configuration of Intel(R) Xeon(R) CPU 3.50 GHz, 64 Gb RAM, and GTX3090 graphics.  Table 3 records the other parameters used in the experiment.

The parameters used for the text noise removal model, construction of features at the part-head level, the fusion of character and word features, and entity relation extraction experiments are listed in Table 4.

### 4.3. Result of Text Noise Removal

**Figure 7:**
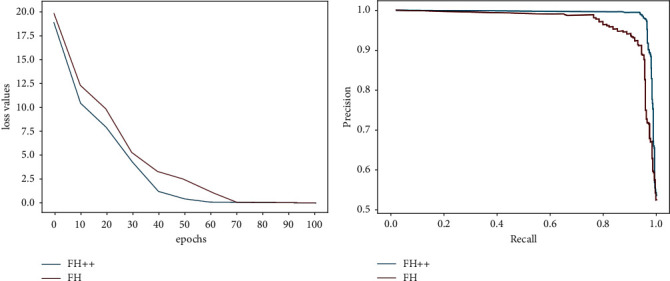
Loss values and PR curves. The blue curve represents the data set FH++, and the red curve represents the data set FH. (a) The curve represents the value of the loss function of the FHER method during the training phase. (b) Curve represents the PR curve based on the training results.

The following are the experimental results of text noise removal, and the model is trained iteratively on the data set FH++.  Figure 7 records the loss values and the final precision/recall curves during the training process, and it can be observed that the loss values gradually decrease, and the model gradually converges as the number of iterative training increases. The precision/recall curves indicate that the model has a good performance.  Table 5 records the precision, recall, and *F*1 values for the text noise removal experiments on FH and the incremental data set FH++. The model can obtain better convergence and accuracy when training with the incremental data set. The positive and negative values in the experimental results shown in this paper are confidence intervals calculated by combining multiple results at a confidence level of 95%.

Table 6 records the experimental results obtained on the FH++ data set where we tried different structures to remove the noise, containing precision, recall, and *F*1 values. In Table 6, both the CNN and CNN-BiLSTM models use the softmax function as the activation function to transform the denoising task into a classification task. The results show that the sequence task using the BiLSTM + CRF model has a better denoising effect.

### 4.4. Result of Entity Relation Extraction

For the entity relation extraction task, Table 7 records the effect of the model on the data set FFH, which contains the values of precision, recall, and *F*1 for a given sentence containing one to five relationships. The experimental results show that the extraction effectiveness of the model decreases as the number of relations in the input sentences increases, which is related to the imbalance of the data itself, with most of the samples in the data set having less than three relations. However, the overall recognition effect is still good.

The FHER method achieves 91.32% accuracy, 82.21% recall, and 86.52% *F*1 value on the FFH data set and achieves good extraction results in few-shot learning.

We did ablation experiments on the proposed denoising model to verify the effectiveness of few-shot learning for denoising. In Table 8, *N* represents the number of relations in the sentence, and the value in the table is the precision values for the entity relation extraction task. The experimental results show a significant decrease in recognition precision after the denoising model for a sentence containing multiple relations because the model identifies group or entity relations that we do not want. Therefore, denoising the input samples in few-shot learning is an effective method.

Similarly, we conducted ablation experiments to add prefix features to enhance and fuse character and word features.  Table 9 records the experimental results. According to the experimental results, richer semantic information can effectively improve the effect of entity relation extraction during few-shot learning. The NIC model represents no features at the constructive radical level in the model, and the NCW model indicates that there are no fused characters and words in the model, and the whole model uses a vector of characters as units.

At last, we test the generalization performance of the method on the data set MDH. The experimental results show that constructing the input at the radical level and fusing characters and words can achieve good results even with smaller data set volumes.  Table 10 records the results of the method FHER on the data set MDH.

### 4.5. Discussion

We discuss the degree of semantic information in the text after its transformation into a radical. There are as many as 3,500 commonly used Chinese characters (http://www.gov.cn/gzdt/att/att/site1/20130819/tygfhzb.pdf). These 3,500 characters can be combined in vast amounts of meaningful words, making the vector space generated after representation learning of Chinese through language models more complex. In contrast, the radicals, which are the main constituents of Chinese characters, can be grouped into 201 radicals for 20,902 Chinese characters (http://www.moe.gov.cn/downloadvideo/yuxinsi/15hanzibushou.zip). Therefore, radicals reduce the complexity of the Chinese vector space.

**Figure 8:**
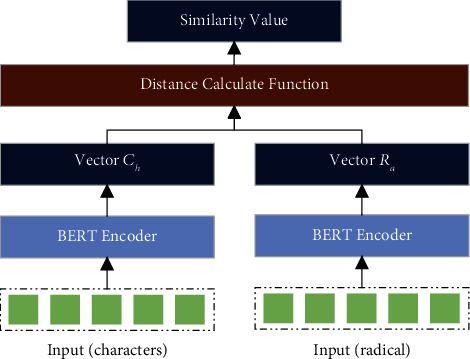
Text similarity calculation structure. The input at the character level and the input at the radical level are transformed by the pretrained model into vectors *C*_*h*_ and *R*_*a*_. The text similarity is obtained by calculating the distance between the vectors *C*_*h*_ and *R*_*a*_.

**Figure 9:**
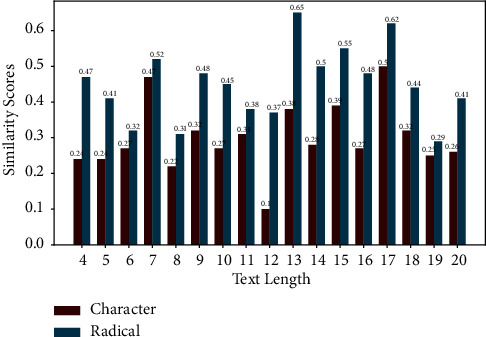
Results of text similarity calculations at the character level and the radical level. The horizontal coordinates represent the sum of the text lengths of the subject and object, while the vertical coordinates represent the similarity results. The blue bars represent radicals, and the red bars represent characters. The text similarity calculated at the radical level is higher than that at the character level.

For the entity groups “olive oil” and “unsaturated fatty acid” and their radical representations, we used the Chinese pretrained language model BERT-wmm to calculate their similarity based on the distance (see Figure 8). We collected subject-object combinations from the data set FFH. The sum of subject and object text lengths ranged from 4 to 20, so text similarity at the character level and the radical level was calculated between each pair of subjects and objects in them.  Figure 9 shows the results of the text-similarity calculation. The experiment proves that the decomposition of Chinese characters into radical representations reduces the complexity of the text to a certain extent.

## 5. Conclusions

This paper proposes a method for entity relation extraction in food health and safety. Three methods are proposed for entity relation extraction under small sample learning to improve the entity relation extraction effect. Firstly, we propose removing text noise according to domain specialization. The experimental results show that removing entity relations in the text unrelated to the domain can significantly improve the effect of entity relation extraction. Then, we construct text features at the radical level by disassembling individual Chinese characters from the characters' structure. The experimental results show that the input at the radical level has low complexity and contains rich semantic features. Finally, we fuse the input text's character vectors and word vectors from the Chinese character constructions. The experimental results show that fusing characters and words can effectively enrich the semantic information in the input vectors. The above method has a good extraction effect in few-shot learning. In the method FHER, the effect of the entity relationship extraction task is subject to a threshold, which leads to an unstable performance of the model. In the future, we will further explore the influence of noise in the text on the degree of deep learning models, the semantic relationships, and differences between the three different levels of Chinese radicals, characters, and words. Moreover, we attempt to transfer the proposed method to other application fields such as agricultural image recognition, greenhouse environmental time-series prediction, and food safety risk assessment [27–31].

## Figures and Tables

**Table 1:** The combination of entity relations present in the text.

Index	Subject	Relation	Object	Definition
1	Enrofloxacin	Belong	The third generation of quinolones	Noise
2	Enrofloxacin	Is	Synthetic broad-spectrum antibacterial drugs	Noise
3	Skin and meat	Contain	Enrofloxacin	Useful
4	The maximum residue limit	Is	100 μg/kg	Useful

**Table 2:** Definition of relationships for the FH data set.

ID	Relation name (CH)		Relation name (EN)	Abbreviations
1		含有	Contains	CO
2		别名	Another name	AN
3		属于	Belong	BE
4		查处	Investigation	IN
5		功效	Efficacy	EF
6		不符合	Noncompliance	NC
7		是为	Is	IS
8		检出	Detection	DE
9		污染物	Contaminants	CT
10		食品类别	Food category	FC
11		不良反应	Adverse reactions	AR
12		无关	Other	O

**Table 3:** Hyperparameter setting.

Hyperparameter	Search space
Bert encoder	Base-uncased BERT-wmm
Bi-LSTM layer	Number of recurrent layers: [2–14, 16, 17]
Batch size	[128, 256, 512, 1024]
Learning rate	[1 × e^−3^, 1 × e^−4^, 1 × e^−5^]
Epochs	[50, 100, 200]
Loss function	Binary cross-entropy Negative log-likelihood
Update strategy	[SGD, Adam]

**Table 4:** Detailed parameters of the experiment.

Model	Construction and output	Training hyperparameters
Text noise removal classifier	Input BERT character Bi-LSTM layer × 8 CRF	Batch size: 128 Learning rate: 0.001 Epochs: 100 Loss function: negative log-likelihood Update strategy: Adam

Feature enhancement	Input Convert function BERT character	—

Chars and words fusion	Input BERT words Combine function sigmoid × 2	Batch size: 128 Learning rate: 0.001 Epochs: 200 Loss function: binary cross-entropy

**Table 5:** Result of text noise removal experiments on data sets FH and FH++.

Data set	*P* (%)	*R* (%)	*F*1 (%)
FH	87.64 ± 0.47	70.12 ± 0.51	77.90 ± 0.53
FH++	**91.12** ± 0.33	**75.34** ± 0.21	**82.48** ± 0.51

**Table 6:** Comparison of noise removal models on data set FH++.

Model	*P* (%)	*R* (%)	*F*1 (%)
CNN	65.34 ± 0.47	33.21 ± 0.31	44.03 ± 0.23
CNN-BiLSTM	78.12 ± 0.33	75.21 ± 0.71	76.63 ± 0.51
BiLTSTM + CRF	**91.12** ± 0.33	**75.34** ± 0.21	**82.48** ± 0.51

**Table 7:** Experimental results for different number relations on data set FFH.

Number of relations	*P* (%)	*R* (%)	*F*1 (%)
*N* = 1	88.64 ± 0.27	63.13 ± 0.31	73.74 ± 0.53
*N* = 2	70.12 ± 0.13	61.24 ± 0.11	65.37 ± 0.31
*N* = 3	55.45 ± 0.42	53.81 ± 0.33	54.61 ± 0.31
*N* = 4	43.10 ± 0.12	50.01 ± 0.09	46.29 ± 1.21
*N* = 5	33.10 ± 0.12	42.01 ± 0.09	37.02 ± 2.21

**Table 8:** Text removal noise ablation experiment results.

Model	*N* = 1 (%)	*N* = 2 (%)	*N* = 3 (%)	*N* = 4 (%)	*N* = 5 (%)
FHER (noise)	85.31 ± 0.87	63.13 ± 0.31	42.74 ± 0.53	21.10 ± 0.32	12.21 ± 0.21
FHER	**88.64** ± 0.27	**70.12** ± 0.13	**55.45** ± 0.42	**43.10** ± 0.12	**33.10** ± 0.12

**Table 9:** Comparison of different degrees of semantic information on data set FFH.

Model	*P* (%)	*R* (%)	*F*1 (%)
NIC	88.91 ± 0.25	81.34 ± 0.86	84.95 ± 0.53
NCW	91.12 ± 0.13	80.24 ± 0.21	85.33 ± 0.31
FHER	**92.32** ± 0.14	**82.21** ± 0.23	**86.97** ± 0.77

**Table 10:** Method generalisation performance experiments on data set MDH.

Model	*P* (%)	*R* (%)	*F*1 (%)
NIC	75.23 ± 0.75	64.14 ± 0.77	69.24 ± 0.81
NCW	73.03 ± 0.86	66.13 ± 0.92	69.40 ± 0.91
FHER	**79.32** ± 0.53	**74.21** ± 0.99	**76.67** ± 0.63

## Data Availability

The FFH data sets used to support the findings of this study are included within the article.
